# Extra- and Intracranial Diffuse Large B-Cell Lymphoma (DLBCL) Mimicking Meningioma: A Case Report and Literature Review

**DOI:** 10.7759/cureus.42500

**Published:** 2023-07-26

**Authors:** Matthias Matejka, Carlos Moreno Beredjiklian, Arwin Rezai, Theo F.J. Kraus, Dominik Pizem, Fritz Klausner, Johannes P. Pöppe, Christoph J. Griessenauer, Christoph Schwartz

**Affiliations:** 1 Neurosurgery, University Hospital Salzburg, Salzburg, AUT; 2 Pathology, University Hospital Salzburg, Paracelsus Medical University, Salzburg, AUT; 3 Neuroradiology, University Hospital Salzburg, Salzburg, AUT

**Keywords:** neurooncology, primary dural lymphoma, meningioma-like lesion, diffuse large b-cell lymphoma, primary central nervous system lymphoma

## Abstract

Primary central nervous system lymphomas (PCNLSs) are malignant non-Hodgkin lymphomas solely affecting the central nervous system (CNS). Here, we present a rare case of extra- and intracranial manifestation without adjacent calvarial infiltration. We report a 67-year-old woman who presented with right leg paresis and hypoesthesia, facial hypoesthesia, focal epileptic seizures, and an indolent tumor on the left parietal scalp. MRI showed a left paramedian extra- and intracranial contrast-enhancing tumor with infiltration of the superior sagittal sinus, but without osseous infiltration on CT. The tumor was radiologically suspected to be a meningioma and resection was performed. Histological examination, however, revealed a diffuse large B-cell lymphoma (DLBCL). Thus, the patient received adjuvant treatment according to the MATRix protocol. We provide a detailed analysis of this rare case with a focus on preoperative radiological findings and differential diagnoses. To the best of our knowledge, this is one of only four published cases of DLBCL with extra- and intracranial manifestation without bone affection.

## Introduction

Primary central nervous system lymphomas (PCNSLs) are highly aggressive malignant non-Hodgkin lymphomas solely found in the central nervous system (CNS), potentially affecting brain parenchyma, spinal cord, dura mater, leptomeninges, cranial nerves, eyes, and cerebrospinal fluid (CSF) [1-2]. PCNSLs are rare, accounting for 3%-4% of all primary brain tumors and 4%-6% of extranodal lymphomas; the incidence is reported to be 0.5 per 100,000 per year [2-3]. Over the last decades, however, the incidence has increased in patients over 60 years, potentially due to demographic changes as well as improved neuroimaging [1]. The median age at diagnosis is 65 years. PCNSLs may affect immunosuppressed as well as immunocompetent patients [2]. Known factors that increase the risk of PCNSL are collagen vascular diseases, acquired immune deficiency syndrome (AIDS), and Epstein-Barr Virus (EBV) infection, among others [1].

## Case presentation

**Figure 1:**
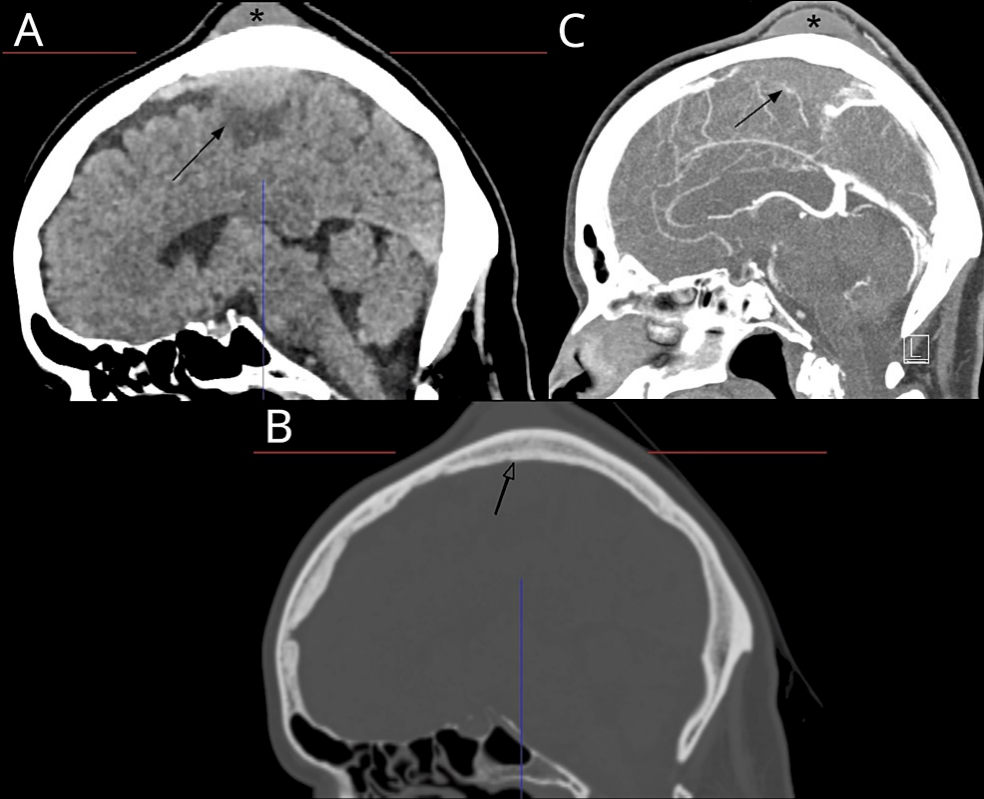
Cranial CT scan. Sagittal CT scan of the head demonstrating an extra- and intracranial mass (asterisk and arrow respectively) (A), sagittal CT head bone window without signs of invasion by the tumor (B), and venous CT angiography showing an obliteration of the superior sagittal sinus due to the intracranial tumor (arrow) (C).

**Figure 2:**
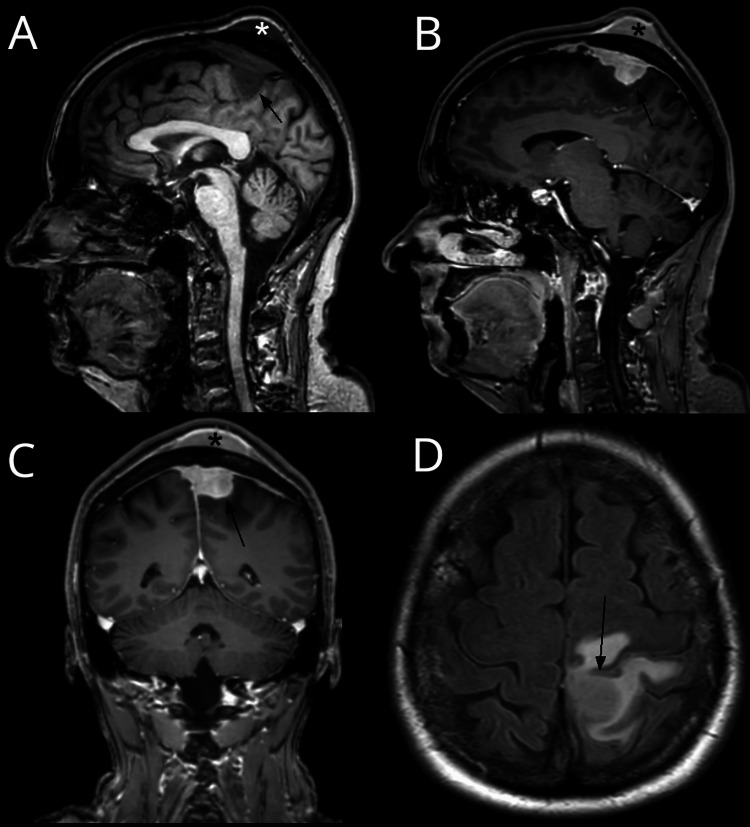
Cranial MRI without and with contrast medium. Sagittal T1-weighted MRI without (A) and with contrast medium (B), as well as coronal T1-weighted MRI with contrast medium showing extra- and intracranial tumor mass (asterisk and arrow respectively), which are hypointense and homogenously contrast enhancing (C). Axial FLAIR-MRI showing the tumor with surrounding edema (D).

A previously healthy 67-year-old woman, with a two-week history of right leg paresis, spastic gait and ataxia, focal epileptic seizures, facial sensation/sensory disturbance, and an indolent tumor on the left parietal scalp was admitted via the neurologic department. On admission, the routine blood work was normal. A cranial CT showed a left-sided, paramedian tumor with an intra- and extracranial component (Figure 1A). Furthermore, complete occlusion of the superior sagittal sinus was confirmed by additional CT-angiogram (Figure 1B). The CT images were initially interpreted to be suspicious for a meningioma, even though no obvious calvarial infiltration was seen (Figure 1C). A consecutively performed MRI also showed an extra- and intracranial tumor with homogenous contrast-enhancement (Figure 2A-D). The patient was scheduled for tumor resection to obtain histopathological diagnosis and to reduce symptomatic brain compression by the tumor mass. The patient gave written informed consent for the procedure.

**Figure 3:**
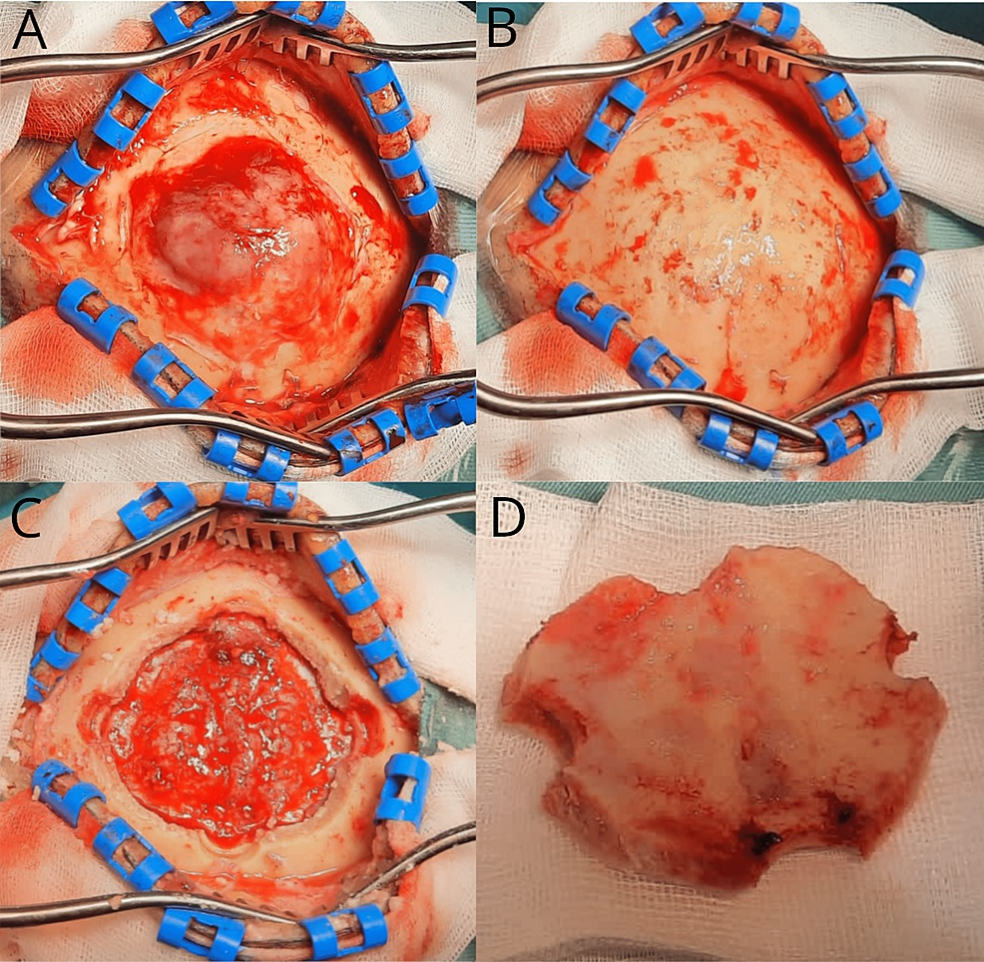
Intraoperative presentation of the extracranial and intracranial tumor mass. Extracranial tumor mass (A) without clear signs of invasion of the underlying calvaria after resection. (B) Removal of the bone showing intracranial tumor and affected dura. (C) Removed bone flap without changes in the bone structure (D).

After skin incision, a subcutaneous rather firm, well-demarcated tumor mass without obvious infiltration of the subcutis was exposed (Figure 3A). This portion of the tumor was easily resected from the underlying calvaria, which appeared intact (Figure 3B). A craniotomy was performed revealing a tumor infiltrated dura beneath (Figure 3C); however, the inner portion of the bone flap did not show any clear tumor infiltration (Figure 3D). The tumor was then resected, and the tumor infiltrated superior sagittal sinus as well as the superior part of the falx were excised. Neither the preoperative imaging nor the intraoperative findings revealed a major diploic vein in the tumor‘s vicinity. It may, however, be hypothesized that smaller emissary veins near the dural lesion may have provided a potential route of tumor cell migration from intra- to extracranial. Since intraoperative frozen sections did not reveal a conclusive histology, it was decided to not reimplant the bone flap, but to perform cranioplasty with a polymethyl methacrylate (PMMA) implant. The postoperative course was uneventful; a postoperative MRI confirmed a gross total resection of the contrast-enhancing tumor volume and the patient continually improved neurologically with complete remission of motor deficits. Histopathological diagnosis of the intra- and extracranial tumor mass as well as the removed bone confirmed a diffuse large B-cell lymphoma (DLBCL). The patient was treated with immuno-chemotherapy according to the MATRix-protocol (rituximab/HD-methotrexate/thiotepa/cytarabine). Two months after surgery and initiation of adjuvant therapy, the patient continually improved neurologically and was independently mobile with a rolling walker.

## Discussion

The presented case is one of very few published cases of patients suffering from a manifestation of DLBCL with an intra- and extracranial tumor component. To the best of our knowledge, only four similar cases have been reported so far (Table 1) [4-7].

**Table 1:** Reported cases of intra- and extracranial manifestation of PDL without bone invasion. DLBCL, diffuse large B-cell lymphoma; CHOP, cyclophosphamide, doxorubicin, vincristine, and prednisolone; WBRT, whole brain radiotherapy

Author (year)	Age (years)/Sex	Location	Surgery	Histology	Adjuvant therapy	Outcome
Tomaszek et al. [4] (1984)	23/male	Right parietal	Biopsy	Hodgkin´s lymphoma	None	Dead (2 years)
Holtas et al. [5] (1985)	60/female	Left frontal	Biopsy	Undifferentiated large cell lymphoma	Steroids	Alive (6 months)
Nishimoto et al. [6] (2003)	63/male	Left parietal	Biopsy	DLBCL	CHOP	Alive
Ochiai et al. [7] (2010)	72/male	Left temporoparietal	Resection	DLBCL	WBRT & CHOP	Alive (1 year)

Tomaszek et al. presented a 23-year-old man with extra- and intracranial lesions in a right parietal location without bone affection on CT. The patient did not have neurological symptoms; therefore, a biopsy of the scalp and epidural masses was performed. Histological examination revealed a Hodgkin lymphoma. No further adjuvant chemotherapy was described. The patient later developed pleural seeding and bone marrow depression and died two years after the initial diagnosis [4]. Holtas et al. reported a case of a 60-year-old woman with an undifferentiated large cell lymphoma with left frontal extra- and intracranial tumors and intact bone on CT. A biopsy was performed. The patient solely received steroids as adjuvant chemotherapy and was alive after 6 months of follow-up [5]. Nishimoto et al. and Ochiai et al. presented 63- and 72-year-old men with left parietal and left temporoparietal extra- and intracranial masses without bone invasion, respectively [6-7]. Both were histologically verified DLBCLs. Nishimoto et al. only performed a biopsy and established subsequent chemotherapy with cyclophosphamide, doxorubicin, vincristine, and prednisolone (CHOP) [6]. After chemotherapy the patient was alive, but the follow-up time was not mentioned. Ochiai et al.´s patient received surgery with gross total resection, adjuvant chemotherapy according to the CHOP regimen, and whole brain radiation therapy (WBRT) [7]. After one year of follow-up, the sensory aphasia had improved [7].

Radiological findings and differential diagnosis

Initial MRI demonstrated an extra-axial paramedian mass in the central region with a broad contact to the dura mater and complete occlusion of the superior sagittal sinus. The lesion was slightly hypointense in T1-weighted sequences, hyperintense in T2-weighted sequences, and showed a dural-tail-sign and a homogenous avid enhancement after the administration of the contrast agent. Based on these imaging characteristics a meningioma was suspected.

In general, intracranial meningiomas account for up to 30% of non-glial tumors and are the most common diagnosis among intracranial neoplasms. Furthermore, the dural attachment with an adjacent dural tail is usually a radiological hallmark feature of intracranial meningiomas found in up to 70% of these tumors; however, when considering differential diagnoses, it has to be remembered that the presence of a dural tail is not exclusively a finding in meningiomas and has been described in 16% of “meningioma-like lesions” [8]. In addition, PCNSLs present as solitary lesions in 60%-70% of the cases, mostly in the hemispheres, basal ganglia, corpus callosum, and periventricular region [1].

Controversially, a second lesion with the same signal characteristics was found extracranially opposite to the first lesion, with no signs of destruction or hyperostotic changes of the calvarium on the CT scan. Bone-infiltrating meningiomas are often associated with hyperostotic, rarely osteolytic changes of the adjacent calvarium [9].

Furthermore, prominent vasogenic brain edema was shown adjacent to the intracranial tumor mass, which can be seen in large meningiomas as well as primary dural lymphomas (PDLs) [7].

**Figure 4:**
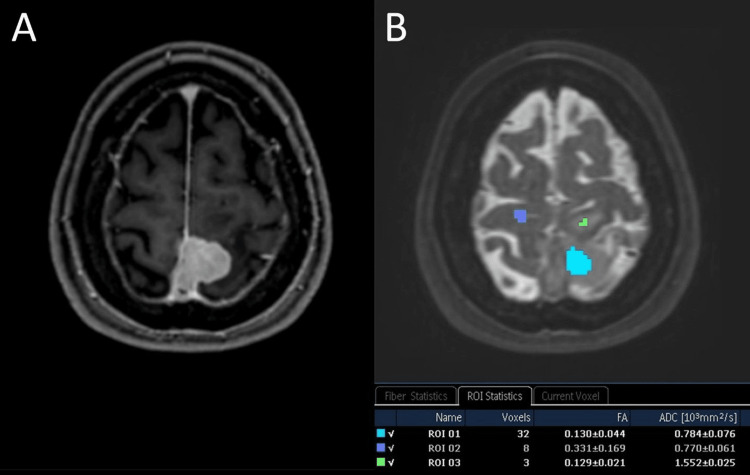
Axial post-contrast T1-weighted image at the level of the intracranial tumor mass (A). Corresponding DTI image with ROIs for the calculation of the FA and ADC values (B): light blue – tumor mass, green – white matter edema, purple – normal gray matter.

The advanced MR-imaging modalities diffusion tensor imaging (DTI) with 32 noncollinear directions and the B-value of 800 s/mm² showed low apparent diffusion coefficient (ADC)-values of 0.784 ± 0.076 10-3 mm2/s as well as low fractional anisotropy (FA) values of 0.130 ± 0.044 in the tumorous mass speaking in favor of a relatively isotropic, highly cellular tumor (Figure 4A-B). MR-spectroscopy was not interpretable because of strong background noise. An alanine peak would have been more specific for meningioma [10]. Important differential diagnostic hallmarks of the PCNSL distinguishing it from meningioma are in general its high cellularity with low ADC values [11-13], a ‘fuzzy’, indistinct brain-tumor interface with absent CSF-cleft-sign [14], as well as a prominent peritumoral vasogenic edema [9]. Perfusion MRI imaging can be beneficial, as lymphomas typically exhibit lower cerebral blood volume (CBV) values than meningiomas [15-16]. In the fluorodeoxyglucose (FDG)-positron emission tomography (PET) lymphomas show avid tracer uptake in comparison to meningiomas, which usually show low activity [17-18].

Histopathology 

**Figure 5:**
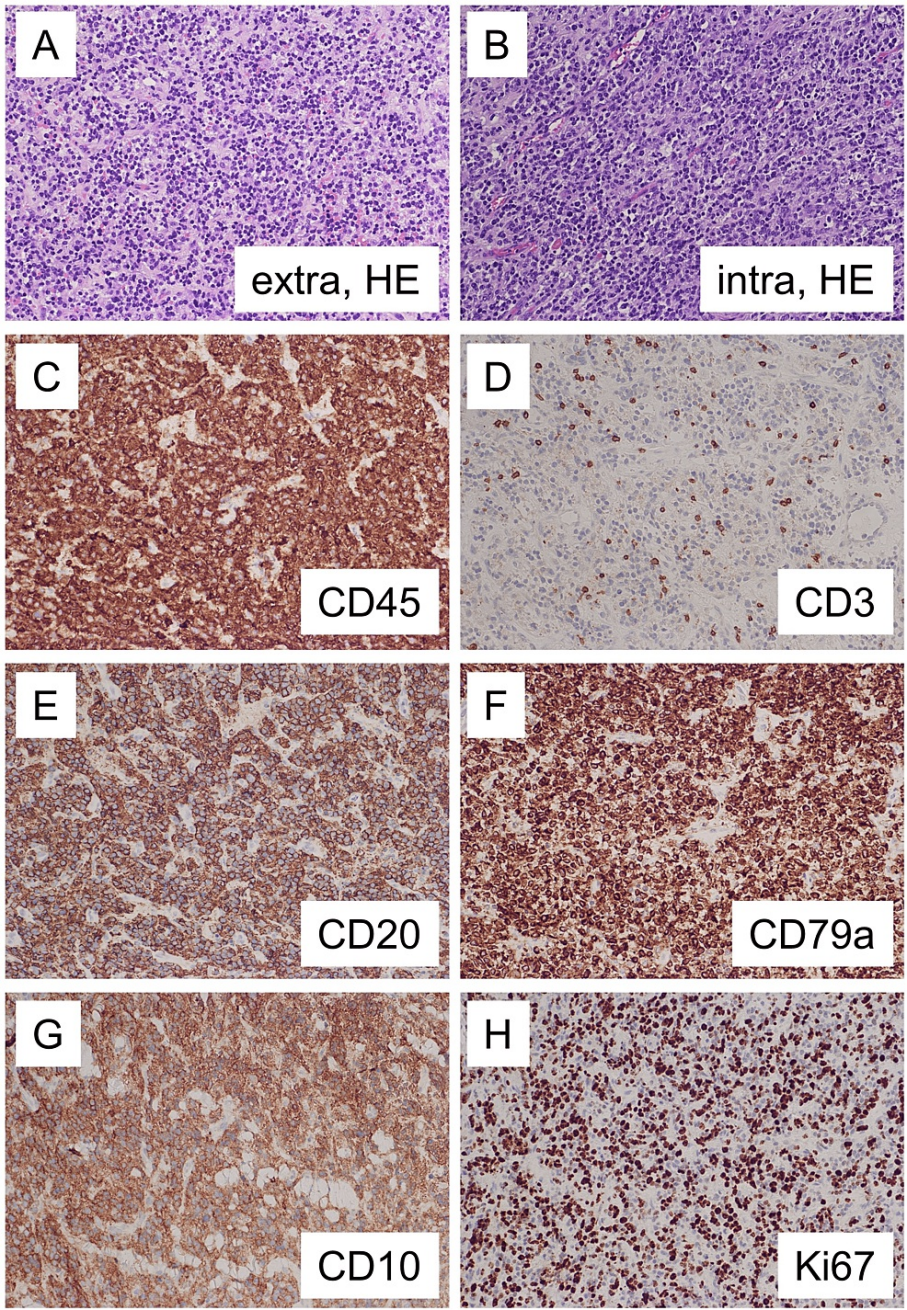
Neuropathological and immunohistochemical analysis of the extracranial and intracranial tumor mass. Neuropathological analysis of HE stained slides showed in both the extracranial (A) and intracranial (B) tumor mass with high cellularity and large tumor cells with large round and ovoid nuclei. Immunohistochemical analysis showed expression of CD45 (C). There were only few CD3 positive T-lymphocytes (D). Nearly all tumor cells expressed CD20 (E), CD79a (F) and CD10 (G). Proliferation was high with 50% Ki67 positive tumor cells (H).

Neuropathological analysis of the tissue obtained showed a tumor with high cellularity. There was diffuse and patternless growth. Tumor cells were large with large round and ovoid nuclei with dense chromatin and distinct nucleoli. These morphological features were found both in extracranial (Figure 5A) and intracranial (Figure 5B) tumor masses. Immunohistochemical analysis showed that tumor cells expressed CD45 (Figure 5C). While there were only a few CD3-positive T-lymphocytes (Figure 5D), tumor cells expressed B-lymphocyte-markers CD20 (Figure 5E) and CD79a (Figure 5F) proving the phenotype of DLBCL. Further classification showed that lymphoma cells expressed CD10 (Figure 5G) resulting in the subtype of germinal center B-cell lymphoma. Ki67 proliferation marker was high with 50% of positive tumor cells (Figure 5H).

Contrary to our suspected diagnosis based on imaging, the histological examination confirmed a PCNSL with the subtype of a primary dural lymphoma (PDL). In a series of 335 patients with PCNSL, only 2.4% were described as PDL [14]. The majority of the histopathological diagnosis of a PDL is a low-grade B-cell marginal zone lymphoma, in contrast to PCNSLs where the diagnosis is usually DLBCL [19].

This case was a rare histopathologically confirmed DLBCL with primary dural manifestation and a second extracranial tumor on the skull. In the literature, there are just a few cases describing an extra- and intracranial PDL diagnosed as DLBCL [20-21]. However, in these cases, the bone of the skull was invaded by lymphoma cells. 

The mechanism of existence of an extra- and intracranial tumor without invading the bone remains unknown. It may be hypothesized that the PDL extended from intracranial to extracranial through emissary veins near the dural lesion.

Treatment and outcome

Immuno-chemotherapy is the hallmark of the treatment of PCNSL patients [2]. The median survival of patients suffering from PCNSL ranges from 12 to 18 months, and for PDL patients a five-year survival rate of 86% has been reported [19]. In most cases, a histological diagnosis can be safely established via biopsy; in our patient, even a biopsy of the extracranial subcutaneous mass would have most likely been sufficient for histopathological diagnosis. Nonetheless, due to the initial interpretation of the available imaging as well as the patient´s neurological symptoms related to the apparent mass effect a microsurgical tumor resection was performed.

Cytoreduction is not widely considered a viable option within the treatment algorithm of PCNSL, and whether tumor resection adds to any potential survival benefits remains highly doubtful [22]. As demonstrated in our case, tumor resection is often only performed if the lesions mimic other pathologies [23]. Nevertheless, recent studies have also shown prolonged survival in patients treated by tumor resection compared to biopsy. Schellekes et al. reported improved survival in patients aged less than 70 years and superficial tumors undergoing resection (35.0 versus 8.9 months, p=0.007) in a retrospective analysis [24]. Furthermore, another study performing a post hoc, “unplanned” analysis showed worse outcomes (progression-free survival p=0.005, overall survival p=0.024) in biopsy patients compared to subtotal/gross total resection cohorts [25]. Rae et al. showed longer survival for patients who underwent resection using three retrospective databases (Table 2) [23]. However, all the above-mentioned literature indicating potential survival benefits from cytoreduction do suffer from significant design flaws, most importantly retrospective/post hoc analyses and selection bias, and no substantial conclusions can be safely drawn from them. Thus, so far, no prospective data on the matter of tumor resection in PCNSL patients exists.

**Table 2:** Median survival in months with 95% CI of biopsy and craniotomy from the three datasets investigated by Rae et al. [24]. The institutional series included patients diagnosed with PCNSL between 2000 and 2017 at their institution. The National Cancer Database is a retrospective dataset sponsored by the American College of Surgeons and the American Cancer Society, constituting 70% of the incidence of invasive cancer cases in the United States. The Surveillance, Epidemiology, and End Results Program is a tumor registry sponsored by the National Cancer Institute, covering approximately 28% of the United States population.

	Institutional series		National cancer database		Surveillance, epidemiology, and end results program		
	Biopsy (n=72)	Craniotomy (n=60)	Biopsy (n=5513)	Craniotomy (n=3423)	Biopsy (n=3350)	Subtotal resection (n=216)	Gross total resection (n=1070)
Median survival (95% CI)	24.7 months (13.8-54.9)	46.0 months (35.7-133.4)	11.0 months (10.1-12.3)	19.5 months (16.8-22.0)	10 months (10-12)	24 months (13-40)	29 months (24-34)

## Conclusions

When planning a tumor resection in a patient with a tumor displaying adjacent extra- and intracranial masses, preoperative imaging must be carefully examined regarding potential differential diagnoses. PCNSL should be kept in mind as they can mimic a meningioma -- as well as any other CNS tumor. Especially, missing bone affection, low ADC values, a ‘fuzzy,’ indistinct brain-tumor interface with absent CSF-cleft-sign, as well as a prominent peritumoral vasogenic edema allude to PCNSL over meningioma.
